# Genomic Differentiation and Diversity in Persian Gulf Hawksbill Turtles (*Eretmochelys imbricata*) Revealed by the First Whole-Genome Sequencing Study

**DOI:** 10.3390/ani16020169

**Published:** 2026-01-07

**Authors:** Mohammadali Farahvashi, Mohammadreza Mohammadabadi, Majid Askari-Hesni, Zeinab Amiri Ghanatsaman, Hojjat Asadollahpour Nanaei

**Affiliations:** 1 Department of Animal Science, Faculty of Agriculture, Shahid Bahonar University of Kerman, Kerman 7616913439, Iran; mafarahvashi@gmail.com; 2 Department of Biology, Faculty of Sciences, Shahid Bahonar University of Kerman, Kerman 7616913439, Iran; mahesni@uk.ac.ir; 3 Animal Science Research Department, Fars Agricultural and Natural Resources Research and Education Center, Agricultural Research, Education and Extension Organization (AREEO), Shiraz 7155863511, Iran; zeynabamiri1237@gmail.com (Z.A.G.); hojat.nanaei@gmail.com (H.A.N.)

**Keywords:** hawksbill turtle, *Eretmochelys imbricata*, whole-genome sequencing, population structure, Persian Gulf

## Abstract

This is the first WGS study of the hawksbill sea turtle (*Eretmochelys imbricata*) from the Persian Gulf. Sequencing of 17 individuals from four nesting islands in the southern part of Iran’s coastline revealed population-level genetic differentiation with consistently low rates of nucleic diversity and differentiated demographic footprints in sampling populations in the Persian Gulf. In a population structure analysis, four populations were discovered; Shidvar had long-term isolation and Nakhiloo demonstrated admixture and higher heterogeneity. ROH and LD profiles are consistent with past bottlenecks, rather than recent inbreeding, and reflect each island’s demographic history. The observed fine-scale genomic divergence (e.g., even between sites in close proximity, such as Ommolgorm and Nakhiloo) suggests that natal homing and local environmental selection are meaningful drivers of genetic differentiation, and that specific conservation strategies should be implemented at each site.

## Simple Summary

Hawksbill sea turtles (*Eretmochelys imbricata*) are critically endangered. The nesting populations in the Persian Gulf are not well studied or described. We sequenced whole genomes from individuals sampled across four islands in the northern Persian Gulf and found genetic differences between turtles from spatially proximate nesting areas, despite having long life spans and overlapping foraging locations. Genetic sampling already shows differences across nesting areas. These differences could be explained by natal homing and environmental heterogeneity in the nesting areas, implying that the conservation of individual nesting beaches is essential and that the loss of one could result in the loss of an entire genetic lineage. Our results thus provide a genomic basis for more informed site-specific conservation in this region.

## 1. Introduction

The Persian Gulf is a semi-enclosed, marginal sea exposed to an arid, subtropical climate. It is surrounded by many of the Earth’s deserts and lies between latitudes 24–30° N and longitudes 48–56° E. The gulf spans about 239,000 km^2^, is approximately 990 km long, and has a maximum width of 370 km [1]. The narrow Strait of Hormuz restricts water exchange between the Persian Gulf and the northern Indian Ocean. Despite this restriction, the Gulf hosts a unique biological community due to its extreme environmental conditions [2]. Species diversity in the Persian Gulf is lower than in other parts of the Indian Ocean, yet endemism is notably high [3]. The Persian Gulf contains some of the northernmost coral reefs in the Indian Ocean, where ancestral genetic diversity is associated with the rapid spread of stress-tolerant coral symbionts in response to Holocene climate change [4]. Corals in the Persian Gulf inhabit a thermally extreme environment, yet symbiont community composition has been shown to remain stable even through episodes of severe bleaching [5], and symbiont fidelity, not flexibility, in the hottest reefs in the world underscores the remarkable resilience of these coral assemblages [6].

In addition to these temperature extremes, salinity levels in the Persian Gulf are unusually high, yet coral growth persists in many areas. However, reefs frequently exhibit signs of stress, including reduced species diversity and widespread partial mortality [7]. At the same time, ancestral genetic diversity has been linked to the rapid spread of stress-tolerant symbionts, providing a mechanism for coral persistence under extreme environmental conditions [4]. The coral reefs of the Persian Gulf hold particular significance in the context of climate change, as present-day thermal conditions already resemble those projected for much of the world’s tropical oceans by the end of the 21st century. Therefore, these corals offer a glimpse into the potential future of global reefs, providing invaluable insights into the limits of thermal adaptation [8,9]. Its shallow, semi-enclosed nature, combined with these extremes, makes the region a valuable natural model for studying climate-driven adaptation.

Within this challenging environment, sea turtles, the reptiles that appeared a long time ago with remarkable evolutionary traits, inhabit the Persian Gulf. Like populations worldwide, they display natal homing (philopatry), undertake long-distance migrations between foraging and nesting grounds, and are characterized by long generation times and relatively low metabolic rates [10,11]. Despite their resilience, global sea turtle populations have declined sharply in recent decades due to exploitation for meat and eggs, commercial trade, incidental capture in fisheries, and the conservation implications of historic sea turtle nesting beach loss [12]. Additional pressures include habitat degradation, coastal development, and climate-related shifts in sex ratios, as sea turtles show temperature-dependent sex determination (TSD), in which low incubation temperatures produce males and high temperatures produce females; hence, there are concerns that climate warming may lead to all-female populations [12,13,14,15]. The International Union for Conservation of Nature (IUCN) Red List classifies sea turtles as ranging from vulnerable to critically endangered. Among sea turtles, the hawksbill is considered the most endangered and is listed as critically endangered [16]. The hawksbill turtle is predominantly distributed in tropical and subtropical waters worldwide, and it nests along various Iranian coasts [17,18]. Hawksbill turtles selectively consume sponges, and by feeding on fast-growing species they act as key regulators of reef biodiversity by reducing competition among sponges. Their absence and low population may decrease sponge and coral diversity [19,20]. Their intricate and irreplaceable ecological role underscores the urgency of recovery efforts, especially in vulnerable regions such as the Persian Gulf.

Genetic diversity is fundamental to the reproductive success and long-term survival of endangered species [21]. Many threatened taxa are confined to small, genetically impoverished, or inbred populations [22], conditions that may increase differentiation while reducing viability [23,24]. Population declines often lead to reduced genetic variation and restricted gene flow [25,26], which can result in inbreeding depression [27] and heightened extinction risk [28]. Assessing genetic diversity and population structure is therefore essential, yet such studies remain limited, and the long-term consequences of disrupted gene flow are poorly understood [29,30]. The level of genetic diversity within a species reflects both the ancestral genetic background and more recent demographic and anthropogenic influences [31]. Historical bottlenecks can drastically reduce effective population size, leaving species vulnerable to inbreeding and genetic erosion [32]. Past climatic shifts and geophysical barriers have also shaped dispersal routes, range dynamics, and gene flow, often driving major changes in population size and genetic composition [33]. Today, human-induced habitat loss and fragmentation represent additional threats, fostering isolation and further erosion of genetic diversity [34,35].

Genomic approaches are increasingly recognized as powerful tools for understanding how genetic drift, mutation, recombination, and natural selection interact to shape the genomes of endangered populations [36]. These methods also enhance conservation outcomes by enabling the identification and management of deleterious variants. Investigating the genetic makeup of hawksbill turtles offers valuable insights into their long-term evolutionary trajectories and provides a clearer understanding of their current population status; genetic analyses have become integral to the development of effective conservation strategies, consistent with conservation genomics of threatened animal species [37].

Traditionally, conservation genetic studies have relied on molecular markers such as mitochondrial control region sequences and nuclear microsatellites [38]. However, such genetic markers may yield incomplete or biased reconstructions of population history, potentially limiting the accuracy of conservation inferences. In contrast, whole-genome sequencing provides high-resolution, genome-wide data that enable an unbiased and comprehensive assessment of genetic diversity—an essential advancement for conservation genetic studies [39].

Given the ecological significance of hawksbill turtles and the conservation challenges they face, genomic analysis of wild populations in understudied and environmentally distinctive regions such as the Persian Gulf is both timely and essential. In this study, we generated and analyzed whole-genome sequencing (WGS) data from hawksbill turtles nesting along the Iranian coast of the Persian Gulf—representing the first such genomic dataset from this wild population.

## 2. Materials and Methods

### 2.1. Sample Collection, DNA Extraction, and Sequencing

**Figure 1:**
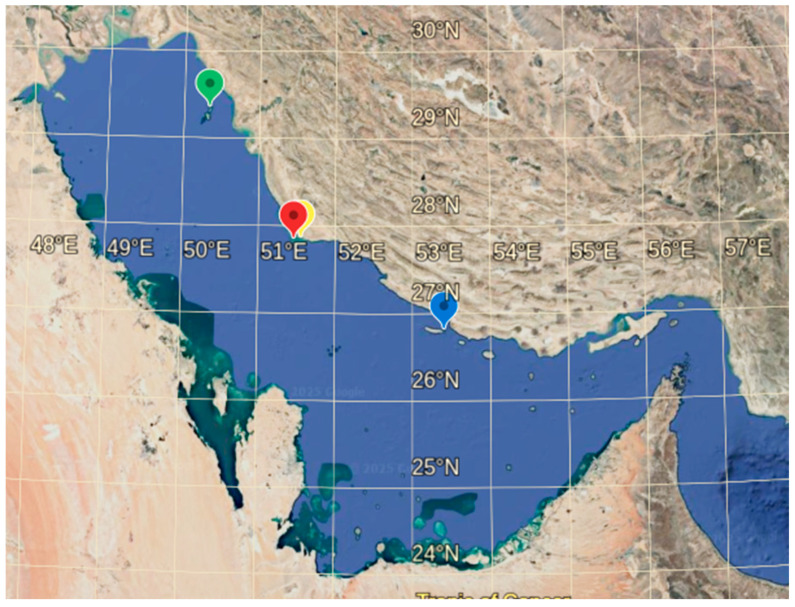
Sampling sites for hawksbill turtles in the northern Persian Gulf: Kharkoo is represented by green, Ommolgorm by yellow, Nakhiloo by red, and Shidvar by blue markers.

Sampling sites are distributed along the southern coasts of Iran in the nesting habitats of the northern Persian Gulf. Sampling was performed in four hawksbill turtle nesting sites including Nakhiloo (N), Kharkoo (K), Ommolgorm (O), and Shidvar (S) Islands (Figure 1). Two small uninhabited islands Nakhiloo and Ommolgorm (Ommolkaram) are located along the coast of Bushehr Province in the northern Persian Gulf. Nakhiloo and Ommolgorm are part of the Dayer-Nakhiloo National Park to the south of the Mond River Delta. Kharkoo Island lies in the northwestern Persian Gulf, approximately 7.3 km from the larger Khark Island. Shidvar Island, an uninhabited island within the Kish Division of Bandar Lengeh County in Hormozgan Province, is located in the northeastern Persian Gulf. All of these islands are designated as national natural monuments [40,41].

Sampling was performed in 2023 between March and June. One sample from each nest was collected on these islands. Samples were taken from nests with emerging hatchlings, and dead hatchlings found during the reproduction season were also collected from various nests. In total, eight samples were taken from Nakhiloo, five from Shidvar, and two each from Ommolgorm and Kharkoo. Following packaging and labeling, all samples were transported on ice and stored immediately at −20 °C.

A section of each sample’s limb was carefully separated and ground into a fine powder using liquid nitrogen. The powdered tissue was then lysed with 500 microliters of a lysis buffer that included proteinase K (200 mg/mL), 100 mM NaCl, 10 mM Tris-HCl, 13 mM EDTA, 35 mM SDS, and 3.5 mM sodium citrate. Total DNA was extracted using chloroform [42]. The concentration and purity of the extracted DNA were assessed both spectrophotometrically with the NanoDrop One device (Thermo Fisher Scientific, Waltham, MA, USA) and through electrophoresis on 1% agarose gel using the Owl™ DuoGel™ Electrophoresis System (Thermo Fisher Scientific, Waltham, MA, USA) and visualized with the Gel Doc™ EZ Imager System (Bio-Rad Laboratories, Hercules, CA, USA).

For each individual, paired-end sequencing libraries were constructed with an average insert size of 318 base pairs. Each library was processed to generate an average read length of 150 base pairs in both directions. Libraries were sequenced using the Illumina NovaSeq instrument at Novogene Bioinformatics Institute, Beijing, China.

### 2.2. Alignment, Genotyping and Variant Calling

FastQC v0.12.1 [43] was used to assess the quality of sequencing reads for each sample. AdapterRemoval v2.3.4 [44] was employed to remove adapter sequences from the raw reads. The trimmed reads were aligned to the *Eretmochelys imbricata* reference genome, generated by Gue et al. [45] and available at https://www.ncbi.nlm.nih.gov/datasets/genome/GCA_030012505.1 (accessed on 1 December 2025). Alignments were performed using the Burrows-Wheeler Aligner (BWA-MEM v0.7.17) with default parameters [46]. SAMtools v1.21 [47] was then used to convert SAM files (.sam) into BAM format (.bam), followed by sorting and indexing. To reduce the risk of false-positive variant calls, Picard Tools v3.4.0 [48] was used to remove potential PCR duplicates, utilizing the MarkDuplicates package.

Mapping quality was assessed using two criteria: (1) the proportion of short reads aligned to the reference genome and (2) the sequencing depth of coverage. These metrics were assessed using the flagstat and depth commands in SAMtools.

For each sample, variant calling and genomic VCF (gVCF) were performed using GATK HaplotypeCaller v4.6.1 [49]; then, the gVCFs were merged by using GATK CombineGVCFs. Joint genotyping was performed with GATK GenotypeGVCFs to generate the final variant call set. Variant types were then classified using GATK SelectVariants, which distinguished single-nucleotide polymorphisms (SNPs) from insertion/deletion variants (INDELs). Quality control and filtering of SNPs were performed using GATK VariantFiltration, excluding any SNPs that did not meet specific quality criteria. Specifically, SNPs were removed if they were not biallelic, had a low confidence score (QUAL < 60), failed the Fisher strand bias test (FS > 60), had poor quality relative to depth (QD < 2), showed low mapping quality (MQ < 40), exhibited extreme discrepancies in mapping quality (MQRankSum < −20), or showed significant positional bias in reads (ReadPosRankSum < −8.0). Variants were also filtered out if they were located in regions with abnormally low or excessively high sequencing coverage (outside 4× the average genome-wide coverage) or within five base pairs of a called INDEL, unless they had a strong confidence score (QUAL > 60).

GATK SelectVariants was used to retain only those variants that met all filtering criteria. The resulting set of high-quality SNPs was considered suitable for downstream genomic analyses.

### 2.3. Genetic Structure and Phylogenetic Inference

To identify the best-fit model of nucleotide substitution for phylogenetic analysis, ModelTest-NG v0.1.7 was used; it compares multiple evolutionary models and ranks them based on statistical criteria such as the Akaike Information Criterion (AIC) and Bayesian Information Criterion (BIC) [50]. Phylogenetic analysis was then performed using IQ-TREE v2.4.0 [51] with the GTR + I + G4 substitution model, as determined by ModelTest-NG. The resulting tree was visualized and annotated using the online tool iTOL (Interactive Tree of Life) v7 [52], enabling interactive exploration of sample relationships; tree topology bootstrap support values were calculated, and the tree was rooted using midpoint rooting.

To mitigate potential bias introduced by tightly linked SNP clusters, linkage disequilibrium (LD)-based pruning was performed using PLINK v1.9.0-b.8 [53] with the parameter --indep-pairwise 50 5 0.2. Principal component analysis (PCA) was conducted using the smartPCA module of the EIGENSOFT package (v6.0beta), with the lsqproject and autoshrink options enabled to improve projection accuracy and reduce bias in eigenvector estimation [54]. The PCA results were visualized in Python v3.12.2 using the Matplotlib v3.9.2 and Pandas v2.2.2 libraries to highlight genetic clustering among samples. Population structure was assessed with ADMIXTURE v1.3 [55] across a range of cluster values (K = 2–6). The optimal number of ancestral populations was determined from cross-validation error and via the Evanno method [56], both implemented and visualized in Python. For each K, admixture proportions were plotted with Matplotlib, with individuals color-coded and labeled according to their predefined population groupings to aid interpretation.

### 2.4. Genetic Diversity and Inbreeding Parameters

To evaluate genetic diversity and individual-level inbreeding, heterozygosity metrics were calculated with VCFtools v0.1.16 [57]. The observed and expected heterozygosity for each sample, together with inbreeding coefficients (F_IS) and using the --het. F_IS values, were displayed as scatter points, providing a clear view of how individual levels of homozygosity deviate from Hardy–Weinberg expectations. Genome-wide nucleotide diversity (π) was estimated in VCFtools using a non-overlapping 100 kb sliding window (--window-pi 100,000). The resulting π values were visualized in Python with the matplotlib and seaborn v0.13.2 libraries. Histograms were used to illustrate the distribution of π across samples, while scatter plots mapped variation along genomic coordinates. To place these results in a broader context, group-level heterozygosity trends were examined, providing insight into overarching patterns of genetic variation.

Runs of homozygosity (ROHs) were identified using PLINK v1.9.0-b.8 with the --homozyg function. ROHs were called using a sliding window of 20 SNPs (--homozyg-window-snp 20), spanning a minimum of 10 kb (--homozyg-kb 10) to reduce the influence of short, potentially uninformative tracts and improve spatial resolution. To maintain stringency and avoid inflated ROH calls in sparsely genotyped regions, we allowed no more than one heterozygous SNP per window (--homozyg-window-het 1). In addition, a minimum density of one SNP per 1000 kb (--homozyg-density 1000) was required to ensure adequate coverage. This parameter configuration was selected to balance sensitivity to shorter homozygous segments with the minimization of false positives. ROH output files were parsed using custom Python scripts to count ROH segments per individual and assess ROH distribution across populations. ROHs were categorized by length as follows: <100 kb, 100–400 kb, and >400 kb. Then, ROH count data were manually structured and visualized using Python’s matplotlib and pandas libraries, grouped as bar charts.

Linkage disequilibrium (LD) decay was performed. LD decay estimation was restricted to the Nakhiloo and Shidvar populations due to sample size constraints. As LD estimation requires a minimum of three individuals per population to yield reliable pairwise genotype correlations [58,59], populations with fewer than three samples, namely Kharkoo and Ommolgorm, were excluded from this analysis. Pairwise linkage disequilibrium (LD) statistics were calculated with VCFtools v0.1.13 using the --geno-r2 option and a maximum inter-SNP distance of 500 kb (--ld-window-bp 500,000). The resulting LD matrices were visualized in R v4.3.3 with ggplot2 v4.0.1, where loess regression curves were fitted to *r*^2^ values plotted against physical distance.

The animal study protocol was approved by the Ethics Committee of Shahid Bahonar University of Kerman (approval date and protocol code available upon request). All experimental protocols and sampling techniques were conducted in accordance with this approval. The ARRIVE 2.0 guidelines (https://arriveguidelines.org/arrive-guidelines (accessed on 3 December 2025)) were followed throughout the study. Since only dead turtle hatchlings were used, no turtles were killed or injured. All sampling procedures involving wild sea turtles complied with relevant national and institutional regulations. Sampling permission was obtained from the Department of Environment of Bushehr Province under permit number 2/1403/1224.

## 3. Results

### 3.1. Mapping Outputs

The average sequencing depth per sample was 24.69X (ranging from 15.59X to 34.95X). After quality filtering, 9,095,246 SNPs were identified across all individuals.

### 3.2. Population Structure Analysis

**Figure 2:**
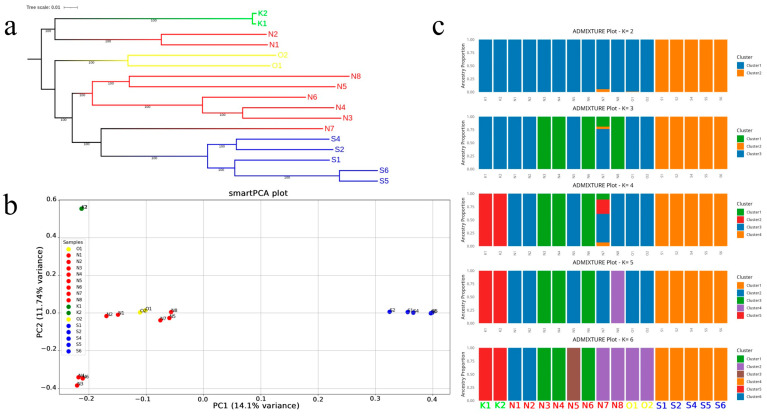
The genetic structure of hawksbill turtles from the northern coasts of the Persian Gulf. (**a**) A maximum likelihood (ML) phylogenetic tree showing the relationships among sampled individuals. The numbers on the branches indicate bootstrap support values. (**b**) Principal component analysis (PCA) of genome-wide SNP variation, with the percentage of variance explained by each of the top two axes indicated in brackets. Note that samples K1 and K2 overlap in the PCA space and are plotted at the same location. (**c**) Admixture analysis illustrating inferred ancestry proportions for K = 2–6, where each vertical bar represents an individual and colors correspond to distinct ancestral clusters.

We reconstructed phylogenetic relationships using a maximum likelihood approach under the best-fit nucleotide substitution model (GTR + I + G4). The resulting topology (Figure 2a) revealed four well-supported clades corresponding to the sampled nesting sites: Kharkoo (green: K1, K2), Ommolgorm (yellow: O1, O2), Nakhiloo (red: N1–N8), and Shidvar (blue: S1–S6). Within Nakhiloo, however, the topology indicates an internal substructure: N1 and N2 formed a distinct subclade, N3–N6 and N8 clustered together, and N7 appeared as a separate lineage. This fine-scale partitioning suggests either historical divergence within the Nakhiloo population or admixture. Despite this internal variation, all Nakhiloo samples clustered more closely with each other than with other populations, supporting their designation as a single regional clade. The Shidvar clade formed a tight and isolated group. Ommolgorm samples formed a distinct clade but clustered more closely with Nakhiloo and Shidvar. Kharkoo, Ommolgorm, and Shidvar each formed distinct clades, indicating moderate divergence from the other populations. Branch lengths reflected genetic distances, with longer branches separating Shidvar from the rest and reinforcing its genetic isolation.

Principal component analysis (PCA; Figure 2b) further highlighted population-level genetic differentiation: the first two principal components captured 14.1% and 11.7% of the variance. On the PCA plot, the Shidvar samples (blue) stand out on the right, separate from the others, forming a distinct cluster that reflects their unique genetic profile. In contrast, Kharkoo samples (green) are grouped in the upper-left quadrant, indicating a differentiated genetic background relative to the other populations. Furthermore, Nakhiloo (red) and Ommolgorm (yellow) samples occupy partially overlapping but distinguishable positions on the left side of the plot, indicating related yet distinct genetic backgrounds. Nakhiloo individuals are broadly distributed across the PCA space, with some clustering near Ommolgorm and others appearing more divergent, suggesting potential substructure or admixture within this population. Consistent with these observations, admixture analysis (Figure 2c) across values of K = 2–6 revealed hierarchical genetic structuring and varying levels of admixture, with patterns that closely mirrored the PCA results and the clustering observed in the phylogenetic tree. At K = 2, Shidvar formed a uniform and exclusive cluster, whereas Ommolgorm, Kharkoo, and Nakhiloo grouped together. At K = 3, Ommolgorm and Kharkoo retained their shared ancestry component from K = 2, which was also present in several Nakhiloo individuals (N1, N2, N5), while others (N3, N4, N6, N8) formed a distinct cluster. At K = 4—identified as the optimal K value based on the Evanno method—Kharkoo samples separated into a distinct cluster, while Ommolgorm remained closely associated with a subset of Nakhiloo individuals. Within Nakhiloo, samples consistently showed elevated levels of mixed ancestry. At K = 5, a finer substructure emerged: Ommolgorm grouped with Nakhiloo individuals such as N1 and N5, while Nakhiloo samples continued to display heterogeneous ancestry profiles. At K = 6, additional clusters were resolved, but the overall structure remained stable. Shidvar samples consistently formed a distinct and homogeneous cluster across all K values, while Ommolgorm appeared as a genetically embedded subset within Nakhiloo rather than as a fully independent cluster. These patterns suggest that Shidvar represents a genetically isolated population, Kharkoo is moderately distinct, and Nakhiloo exhibits internal substructure and admixture, with Ommolgorm likely reflecting localized lineage within the broader Nakhiloo population. When considered together, the maximum likelihood phylogenetic tree, PCA clustering, and admixture ancestry proportions consistently highlight population-associated genetic differentiation. Kharkoo formed both a separate clade and a distinct ancestry component, yet retained genetic affinity with Nakhiloo and Ommolgorm. Nakhiloo showed the greatest internal diversity, with clear evidence of admixture reflected in the heterogeneous ancestry profiles and dispersed PCA positions. The close genetic relationship between Ommolgorm and Nakhiloo was reinforced across all analyses, pointing to shared ancestry or recent divergence. In contrast, Shidvar consistently appeared as a cohesive and isolated population, forming a distinct clade in the phylogenetic tree, a separate cluster in the PCA, and a uniform admixture profile across all tested K values.

### 3.3. Genomic Diversity and Inbreeding

**Figure 3:**
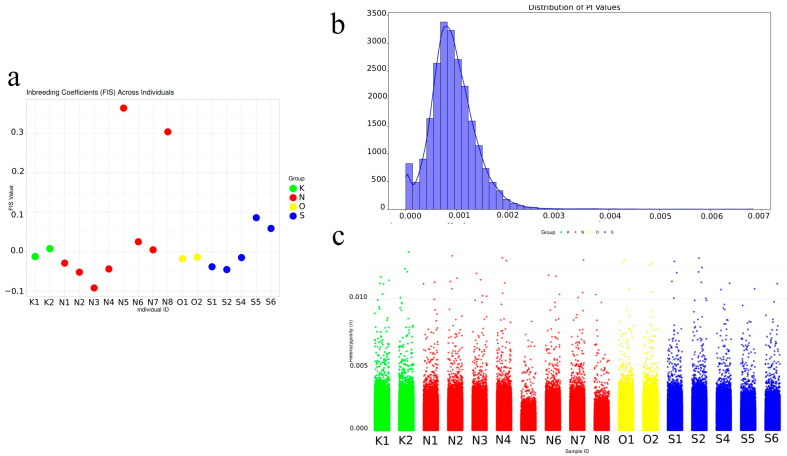
Genomic diversity and inbreeding in hawksbill turtles. (**a**) Inbreeding coefficients (F_IS). (**b**) Genome-wide nucleotide diversity (π) calculated across non-overlapping 100 kb windows. (**c**) Per-sample genomic heterozygosity (π) plots of window-based estimates.

Inbreeding coefficients (F_IS) calculated using VCFtools --het revealed values ranging from −0.0919 to +0.3638 across individuals (Table S1). The majority of samples (15 of 17) exhibited F_IS values between −0.09 and +0.08, consistent with Hardy–Weinberg expectations and indicating no evidence of recent inbreeding. Two individuals (N5 and N8) showed elevated F_IS values (>0.3), suggestive of moderate inbreeding or possible technical artifacts. These patterns are illustrated in Figure 3a, which presents individual F_IS values as a scatter plot.

Genome-wide nucleotide diversity (π) was generally low, as reflected in the distribution of window-based π estimates (Figure 3b). The distribution was strongly left-skewed, with a modal peak near 0.001, suggesting limited genetic diversity across the genome—pattern consistent with small or isolated populations. Per-sample windowed heterozygosity estimates (Figure 3c) reinforced this trend, showing uniformly low π values across individuals and populations.

**Figure 4:**
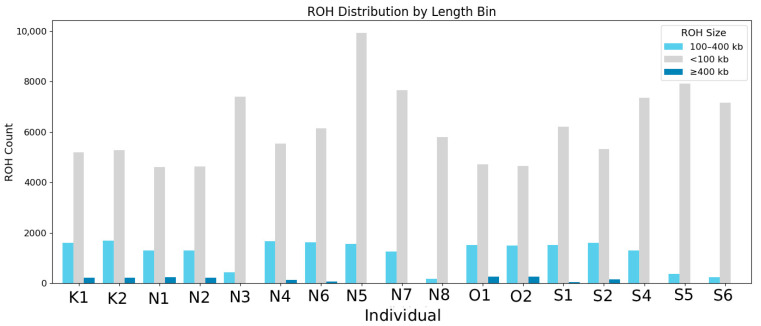
Per-individual distribution of runs of homozygosity (ROHs) by length category.

ROH analysis (Table S2; Figure 4) revealed a predominance of short tracts (<100 kb) across all individuals, with counts ranging from 4611 in N1 to 9926 in N5. Intermediate ROHs (100–400 kb) occurred at moderate frequencies, typically between 244 in S6 and 1690 in K2. Long ROHs (>400 kb) were rare, with most individuals exhibiting fewer than 260 tracts. Nakhiloo turtles displayed the widest range of ROH counts, with N5 exhibiting the highest number of short ROHs and N8 showing no long ROHs. Kharkoo (K1, K2) and Ommolgorm (O1, O2) individuals displayed relatively balanced counts of short and intermediate ROHs, whereas Shidvar turtles (S1–S6) consistently exhibited high numbers of short ROHs and very few long tracts.

**Figure 5:**
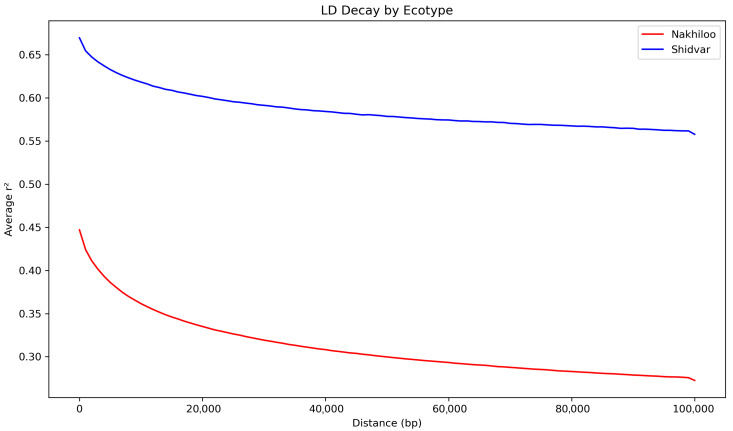
LD decay patterns in Nakhiloo and Shidvar populations.

Linkage disequilibrium (LD) decay patterns (Figure 5) differed markedly between the Nakhiloo and Shidvar populations. Across the genome, Shidvar consistently exhibited higher average *r*^2^ values than Nakhiloo. Moreover, LD decay was substantially slower in Shidvar, whereas Nakhiloo showed a steeper decline in *r*^2^ with increasing physical distance.

Collectively, these findings provide a broad perspective on genomic diversity and inbreeding across the studied populations. Overall, genome-wide diversity was low, with minimal evidence of recent inbreeding, yet clear population-level patterns emerged in homozygosity and linkage disequilibrium. The predominance of short ROHs, together with consistently low nucleotide diversity and heterozygosity across individuals, points to a conserved genetic background. At the same time, elevated F_IS values in certain individuals and contrasting LD decay dynamics between Nakhiloo and Shidvar underscore subtle variation both within and across populations.

## 4. Discussion

This study presents the first whole-genome sequencing (WGS) analysis of hawksbill sea turtles (*Eretmochelys imbricata*) from the Persian Gulf, offering unprecedented resolution into the population genomics of this critically endangered species in a region characterized by extreme environmental gradients. By analyzing 17 individuals from four nesting islands along the southern Iranian coast, we uncovered pronounced population-level genetic differentiation, uniformly low genome-wide diversity, and distinct demographic signatures. These findings refine and extend earlier microsatellite- and mitochondrial-based studies [60,61,62,63] and underscore the value of high-resolution genomic data for conservation planning in understudied marine systems.

### 4.1. Population-Level Genetic Structure

Phylogenetic reconstruction, principal component analysis (PCA), and admixture consistently identified four distinct, population-specific genetic clusters. Shidvar formed a tight, long-branched, and homogeneous clade, while Nakhiloo exhibited broad dispersion and mixed ancestry. These genome-wide patterns corroborate earlier reports of regional differentiation based on microsatellite and mtDNA markers [60,61,62,63], but provide finer-scale resolution of substructure and admixture, particularly within Nakhiloo and Ommolgorm. Comparable fine-scale partitioning has been documented in Indonesia [64], driven by localized nesting habitats and restricted gene flow. The genetic proximity between Ommolgorm and Nakhiloo suggests recent divergence or ongoing connectivity, while Kharkoo forms a moderately distinct cluster. Shidvar’s long branch lengths—especially for individuals S5 and S6—along with uniform ancestry across all K values, indicate long-term isolation and minimal gene flow.

The pronounced genetic differentiation among geographically proximate nesting sites, despite potential overlap in adult foraging areas, is best explained by the interplay of early-life dispersal and strong natal homing [10,65]. After hatching, juveniles enter a pelagic phase shaped by ocean currents and gyres [66,67], yet geomagnetic imprinting enables adult females to return to natal beaches [68]. Environmental gradients within the Gulf such as salinity, temperature, and pollution may impose differential selective pressures [69,70], reinforcing genetic divergence even when foraging areas overlap [71].

### 4.2. Genetic Diversity and Inbreeding

Genome-wide nucleotide diversity (π) and per-sample heterozygosity were uniformly low, with a left-skewed π distribution peaking near 0.001. These patterns are consistent with small effective population sizes and historical bottlenecks [62,72]. While most individuals conformed to Hardy–Weinberg expectations, two Nakhiloo samples (N5, N8) showed elevated inbreeding coefficients (F_IS > 0.3), a pattern that has raised conservation concerns in other hawksbill populations (e.g., [73]). ROH analysis revealed a predominance of short tracts (<100 kb), suggesting ancient demographic contractions rather than recent consanguinity [74]. Comparative studies in mammals and livestock support this interpretation [75,76]. Unlike the high inbreeding reported in Singapore hawksbills [73], Persian Gulf populations appear genetically depauperate but not recently inbred, as suggested by the predominance of short ROHs [74].

Population-specific LD decay profiles further illuminate demographic contrasts. Shidvar exhibited slower LD decay and higher mean *r*^2^ values, consistent with reduced historical recombination and long-term small effective population size. Nakhiloo showed steeper LD decay, suggesting higher recombination and more dynamic demographic history. These patterns resemble the genomic footprints of long-term decline observed in other taxa [33,77].

### 4.3. Conservation Implications and Recommendations

Hawksbill turtles are highly sensitive to climate change, particularly through temperature-dependent sex determination and coral reef degradation. Rising sand temperatures skew hatchling sex ratios and may reduce hatching success [13,14,15]. Shidvar’s low diversity and limited recombination suggest reduced adaptive capacity, while Nakhiloo’s heterogeneity may buffer against environmental change. Recent genomic studies emphasize the importance of preserving adaptive potential through the protection of genetically diverse rookeries [62,64,73].

The genomic distinctiveness and low diversity of Persian Gulf hawksbills highlight urgent conservation needs. Shidvar, as a genetically isolated and vulnerable population, requires site-specific management and strict disturbance minimization. Nakhiloo functions as a regional connectivity hub and genetic reservoir. Conservation efforts should preserve its internal diversity and monitor structural shifts, consistent with broader conservation genomics evidence that highlights the critical role of genetic diversity in maintaining resilience under climate change [64,73]. Empirical studies of hawksbill populations further demonstrate that rookeries act as reservoirs of genetic variation and hybridization, reinforcing the need for site-specific management strategies [78].

The pronounced genomic differentiation observed among nesting sites including between geographically close rookeries such as Ommolgorm and Nakhiloo underscores the importance of protecting each site as a distinct evolutionary unit. Despite the brief time hatchlings spend at nesting beaches and the long maturation period, strong natal homing ensures that females return to their birthplaces to nest. Importantly, adult female hawksbill turtles spend only brief periods at nesting beaches for oviposition, while their foraging habitats are widely shared and overlapping across the Gulf, confirming that genetic divergence arises from nesting site fidelity rather than differences in foraging grounds [68,71]. This fidelity maintains a fine-scale genetic structure over ecological timescales, even in the absence of physical barriers. Across all sampled nesting sites, distinct genomic signatures were detected despite their geographic proximity, suggesting that even subtle differences in oceanographic drift, geomagnetic imprinting, or nesting microhabitats may contribute to divergence [68]. Future work integrating genomic data with telemetry and ecological observations will be essential to fully resolve connectivity among Persian Gulf nesting sites, complementing the genomic differentiation patterns revealed here. The loss of any single nesting site could result in the irreversible loss of unique genetic variants or locally adapted lineages, reducing the species’ overall evolutionary potential. This highlights the need for site-specific conservation strategies that recognize the biological significance of even small or marginal rookeries.

As climate change and coastal development intensify, maintaining genetic resilience will be essential. Future strategies should integrate genomic monitoring with ecological and telemetry data, and screen for hybridization and deleterious variation.

## 5. Conclusions

This study provides the first WGS-based assessment of hawksbill turtles in the Persian Gulf, revealing population-level genetic differentiation, low genome-wide diversity, historical bottlenecks but no widespread recent inbreeding, and distinct demographic signatures. The genetic isolation of Shidvar appears as the most striking observation, underscoring the need for site-specific conservation efforts. As Kharkoo and Ommolgorm were represented by only two individuals each, interpretations for these sites should be made cautiously; nevertheless, their consistent clustering still points to meaningful differentiation. Together, these findings provide a broader genomic vision of Persian Gulf hawksbill turtles and a foundation for targeted conservation strategies in a rapidly changing region.

## Data Availability

The data can be provided by the corresponding author on reasonable request.

## Supplementary Materials

The following supporting information can be downloaded at: https://www.mdpi.com/article/10.3390/ani16020169/s1, Table S1: Observed (O) and expected (E) homozygosity, number of genotyped sites, and inbreeding coefficient (F) per individual; Table S2: Counts of runs of homozygosity (ROHs) per individual by length category.
