# Supplementary material for: Genomic Differentiation and Diversity in Persian Gulf Hawksbill Turtles (Eretmochelys imbricata) Revealed by the First Whole-Genome Sequencing Study
Source: Animals (Basel). 2026 Jan 7;16(2):169. doi: 10.3390/ani16020169 (PMC12837703; doi:10.3390/ani16020169)
Supplement: Supplementary file 1 [file animals-16-00169-s001.zip › animals-4055943-supplementary-Table S1-edited.pdf]

Table S1. Observed (O) and expected (E) homozygosity, number of genotyped sites, and inbreeding coefficient (F) per individual.

Individual	O(HOM)	E(HOM)	N-Sites	F
K1	5219950	5247437.5	7480042	0.01231
K2	5262143	5245240.9	7475409	0.00758
N1	5121147	5184490.4	7383067	-0.02881
N2	5111543	5226824.1	7447516	-0.05191
N3	4865896	5062946.4	7206272	-0.09194
N4	5136243	5233758.6	7461002	-0.04378
N5	5137229	4463555.2	6315341	0.36380
N6	5122291	5068306.2	7214348	0.02516
N7	5024154	5014268.6	7137486	0.00466
N8	4466494	3962391.0	5622220	0.30371
O1	5197306	5237417.9	7463725	-0.01802
O2	5171337	5201809.1	7412541	-0.01378
S1	5078707	5162601.5	7355037	-0.03827
S2	5149326	5250526.3	7485051	-0.04529
S4	5036851	5068885.9	7216482	-0.01492
S5	4699897	4535800.9	6441141	0.08612
S6	4598998	4487650.8	6375931	0.05897

Observed and expected counts of homozygous genotypes, total number of genotyped sites, and estimated inbreeding coefficient (F) per individual. Positive F indicates excess homozygosity relative to Hardy–Weinberg expectations.
