# Supplementary material for: Genomic Differentiation and Diversity in Persian Gulf Hawksbill Turtles (Eretmochelys imbricata) Revealed by the First Whole-Genome Sequencing Study
Source: Animals (Basel). 2026 Jan 7;16(2):169. doi: 10.3390/ani16020169 (PMC12837703; doi:10.3390/ani16020169)
Supplement: Supplementary file 1 [file animals-16-00169-s001.zip › animals-4055943-supplementary-Table S2-edited.pdf]

Table S2. Counts of runs of homozygosity (ROHs) per individual by length category.

Individual	100-400 kb	<100 kb	>400 kb
K1	1598	5202	229
K2	1690	5287	218
N1	1314	4611	256
N2	1316	4627	226
N3	451	7394	2
N4	1667	5541	134
N6	1635	6158	63
N5	1566	9926	16
N7	1270	7670	5
N8	184	5796	0
O1	1519	4713	263
O2	1508	4654	259
S1	1519	6213	55
S2	1598	5326	163
S4	1313	7365	11
S5	376	7932	0
S6	244	7166	0

Counts of runs of homozygosity (ROHs) identified in each individual, grouped into three length categories: short (<100 kb), medium (100–400 kb), and long (>400 kb). Elevated counts of long ROHs indicate more recent inbreeding, whereas abundant short ROHs reflect older or persistent background homozygosity.
